# *tert*-Butyl 3-(8-bromo-4*H*,10*H*-1,2-oxazolo[4,3-*c*][1]benzoxepin-10-yl)-2-methyl-1*H*-indole-1-carboxyl­ate

**DOI:** 10.1107/S1600536810027297

**Published:** 2010-07-17

**Authors:** Ankur Trigunait, P. Malathy, K. Ramachandiran, P. T. Perumal, K. Gunasekaran

**Affiliations:** a Centre of Advanced Study in Crystallography and Biophysics, University of Madras, Guindy Campus, Chennai 600 025, India; b CLRI, Adyar, Chennai 600 020, India

## Abstract

In the title compound, C_25_H_23_BrN_2_O_4_, the seven-membered ring adopts a twisted-boat conformation. The indole ring system is planar within 0.021 (2) Å and the ester group [–C(=O)—O—C–] is almost coplanar with it [dihedral angle = 3.0 (2)°]. The conformation of the ester group is influenced by intra­molecular C—H⋯O inter­actions. In the crystal structure, mol­ecules are linked into chains along the *b* axis by C—H⋯N hydrogen bonds.

## Related literature

For general background to and biological applications of nitro­gen- and oxygen-containing heterocyclic compounds, see: Furstner (2003 ▶); Liddell (2002 ▶); Caramella & Grunanger (1984 ▶); Stormer *et al.* (2004 ▶); Erdelyi *et al.* (2008 ▶). Hou *et al.* (2003 ▶). For puckering parameters, see: Cremer & Pople (1975 ▶). For asymmetry parameters, see: Nardelli (1983 ▶).
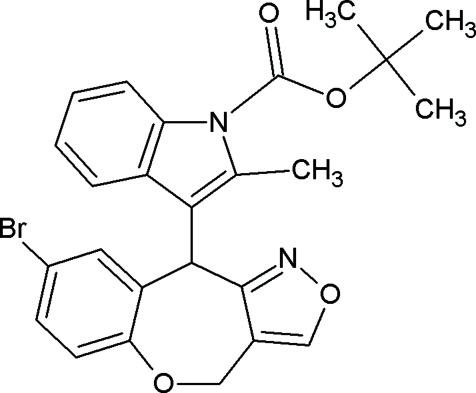


## Experimental

### 

#### Crystal data

C_25_H_23_BrN_2_O_4_
*M* *_r_* = 495.36
Monoclinic, 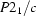

*a* = 16.0494 (6) Å
*b* = 9.6497 (4) Å
*c* = 16.2202 (7) Å
β = 116.267 (2)°
*V* = 2252.66 (16) Å^3^
*Z* = 4
Mo *K*α radiation
μ = 1.86 mm^−1^
*T* = 293 K
0.20 × 0.20 × 0.20 mm

#### Data collection

Bruker SMART APEXII area-detector diffractometer
Absorption correction: multi-scan (*SADABS*; Bruker, 2008 ▶) *T*_min_ = 0.982, *T*_max_ = 0.982
20668 measured reflections
5584 independent reflections
2780 reflections with *I* > 2σ(*I*)
*R*_int_ = 0.049

#### Refinement

*R*[*F* ^2^ > 2σ(*F* ^2^)] = 0.046
*wR*(*F* ^2^) = 0.134
*S* = 1.00
5584 reflections
289 parameters
1 restraint
H-atom parameters constrained
Δρ_max_ = 0.40 e Å^−3^
Δρ_min_ = −0.48 e Å^−3^

### 

Data collection: *APEX2* (Bruker, 2008 ▶); cell refinement: *SAINT* (Bruker, 2008 ▶); data reduction: *SAINT*; program(s) used to solve structure: *SHELXS97* (Sheldrick, 2008 ▶); program(s) used to refine structure: *SHELXL97* (Sheldrick, 2008 ▶); molecular graphics: *ORTEP-3* (Farrugia, 1997 ▶); software used to prepare material for publication: *SHELXL97* and *PLATON* (Spek, 2009 ▶).

## Supplementary Material

Crystal structure: contains datablocks global, I. DOI: 10.1107/S1600536810027297/ci5101sup1.cif

Structure factors: contains datablocks I. DOI: 10.1107/S1600536810027297/ci5101Isup2.hkl

Additional supplementary materials:  crystallographic information; 3D view; checkCIF report

## Figures and Tables

**Table 1:** Hydrogen-bond geometry (Å, °)

*D*—H⋯*A*	*D*—H	H⋯*A*	*D*⋯*A*	*D*—H⋯*A*
C14—H14*A*⋯O3	0.96	1.93	2.694 (4)	135
C24—H24*B*⋯O3	0.96	2.37	2.961 (5)	120
C11—H11⋯N1^i^	0.93	2.53	3.404 (4)	156

Symmetry code: (i) 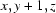.

**Fig. 1.**
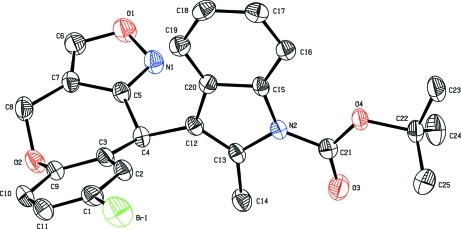
Molecular structure of the title compound, showing the atom-numbering scheme and 30% probability displacement ellipsoids. H atoms have been omitted for clarity.

**Fig. 2.**
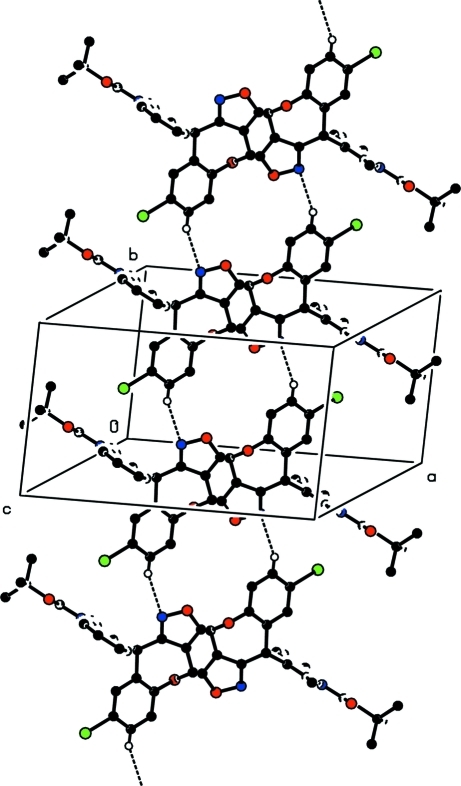
The crystal packing of the title compound. H atoms not involved in hydrogen bonding (dashed lines) have been omitted for clarity.

## supplementary crystallographic information

### Comment

Nitrogen and oxygen containing heterocycles are ubiquitous substructures in myriad of biologically active natural products and small-molecule pharmaceuticals (Furstner, 2003; Liddell, 2002). The nitrile oxide cycloaddition is a useful method to prepare heterocyclic compounds (Caramella & Grunanger, 1984). Isoxazole, the cycloadduct of nitrile oxide, is regarded as a versatile synthetic precursor for γ-amino alcohols and β-hydroxy ketones. Isoxazoles are found in some natural products, such as ibotenic acid. Ibotenic acid is naturally occurring in mushrooms *Amanita muscaria* and *Amanita pantherina*. Ibotenic acid is a powerful neurotoxin that is used as a brain-lesioning agent and has shown to be highly neurotoxic when injected directly into the brains of mice and rats. Isoxazoles also form the basis for a number of drugs, including the COX-2 inhibitor valdecoxib. Valdecoxib is a prescription drug used in the treatment of osteoarthritis, rheumatoid arthritis, painful menstruation and menstrual symptoms (Stormer *et al.*, 2004; Erdelyi *et al.*, 2008).

In the title (Fig. 1), the indole ring system is planar within ±0.021 (2) Å. The oxepane ring adopts a twisted boat conformation with puckering parameters (Cremer & Pople, 1975) and asymmetry parameters (Nardelli, 1983) of q_2_ = 0.654 (3) Å, φ_2_ = 179.9 (3)°, q_3_ = 0.380 (3) Å, φ_3_ = 222.4 (3)° and Δ_s_(C7) = 34.3 (3)°. The position of atom O2 which lies between bromobenzene and isooxazone rings is defined by torsion angles O2—C8—C7—C5 of -34.0 (4)° and O2—C9—C10—C11 of 176.4 (3)°.

The ester group [-C(═O)-O-C-] is coplanar with the indole ring system [dihedral angle 3.0 (2)°]. The planarity is facilitated by intramolecular C14—H14A···O3 C24—H24B···O3 hydrogen bonds (Table 1). A free rotation about the O4—C22 single bond [1.492 (3) Å] is restricted by the C24—H24B···O3 hydrogen bond. The angles around atom C4 [C12—C4—C3 = 113.6 (2)° and C5—C4—C3 = 115.6 (2)°] deviates significantly from ideal tetrahedral values which may be as a result of steric interactions between isooxazole, bromophenol and indole groups.

Atom C11 in the molecule at (x,y,z) acts as donar to atom N1 at (x, 1+y, z), forming a chain running along the *b* axis (Fig. 2).

### Experimental

[3-(5-Bromo-2-prop-2-ynyloxy-phenyl)-2-nitro-ethyl]-2-methyl-1H-indole] (1.0 mmol) and N,N-dimethyl-4-aminopyridine (0.2 mmol) were dissolved in toluene (5 ml). Di-tert-butyl dicarbonate (2.5 mmol) in toluene (5 ml) was added in portions over a period of 0.5 h at 363 K to the nitroalkane solution and the reaction was allowed to proceed for a further 2 h. The mixture was evaporated and the product was purified by column chromatography using ethyl acetate-petroleum ether (2:8) as eluent. Single crystals appeared from the same eluent mixture.

### Refinement

H atoms were positioned geometrically (C–H = 0.93–0.98 Å) and allowed to ride on their parent atoms, with U_iso_(H) = 1.5U_eq_(C) for methyl H and 1.2U_eq_(C) for other H atoms.

### Crystal data

**Table:**

C_25_H_23_BrN_2_O_4_	*F*(000) = 1016
*M**_r_* = 495.36	*D*_x_ = 1.461 Mg m^−^^3^
Monoclinic, *P*2_1_/*c*	Mo *K*α radiation, λ = 0.71073 Å
Hall symbol: -P 2ybc	Cell parameters from 1964 reflections
*a* = 16.0494 (6) Å	θ = 1.4–28.4°
*b* = 9.6497 (4) Å	µ = 1.86 mm^−^^1^
*c* = 16.2202 (7) Å	*T* = 293 K
β = 116.267 (2)°	Block, colourless
*V* = 2252.66 (16) Å^3^	0.20 × 0.20 × 0.20 mm
*Z* = 4	

### Data collection

**Table:**

Bruker SMART APEXII area-detector diffractometer	5584 independent reflections
Radiation source: fine-focus sealed tube	2780 reflections with *I* > 2σ(*I*)
graphite	*R*_int_ = 0.049
ω and φ scans	θ_max_ = 28.4°, θ_min_ = 1.4°
Absorption correction: multi-scan (*SADABS*; Bruker, 2008)	*h* = −21→21
*T*_min_ = 0.982, *T*_max_ = 0.982	*k* = −12→12
20668 measured reflections	*l* = −21→17

### Refinement

**Table:**

Refinement on *F*^2^	Primary atom site location: structure-invariant direct methods
Least-squares matrix: full	Secondary atom site location: difference Fourier map
*R*[*F*^2^ > 2σ(*F*^2^)] = 0.046	Hydrogen site location: inferred from neighbouring sites
*wR*(*F*^2^) = 0.134	H-atom parameters constrained
*S* = 1.00	*w* = 1/[σ^2^(*F*_o_^2^) + (0.0504*P*)^2^ + 1.2404*P*] where *P* = (*F*_o_^2^ + 2*F*_c_^2^)/3
5584 reflections	(Δ/σ)_max_ = 0.002
289 parameters	Δρ_max_ = 0.40 e Å^−^^3^
1 restraint	Δρ_min_ = −0.47 e Å^−^^3^

### Special details

**Table:**

Geometry. All esds (except the esd in the dihedral angle between two l.s. planes) are estimated using the full covariance matrix. The cell esds are taken into account individually in the estimation of esds in distances, angles and torsion angles; correlations between esds in cell parameters are only used when they are defined by crystal symmetry. An approximate (isotropic) treatment of cell esds is used for estimating esds involving l.s. planes.
Refinement. Refinement of F^2^ against ALL reflections. The weighted R-factor wR and goodness of fit S are based on F^2^, conventional R-factors R are based on F, with F set to zero for negative F^2^. The threshold expression of F^2^ > 2sigma(F^2^) is used only for calculating R-factors(gt) etc. and is not relevant to the choice of reflections for refinement. R-factors based on F^2^ are statistically about twice as large as those based on F, and R- factors based on ALL data will be even larger.

### Fractional atomic coordinates and isotropic or equivalent isotropic displacement parameters (Å^2^)

**Table:**

	*x*	*y*	*z*	*U*_iso_*/*U*_eq_	
Br1	0.21016 (4)	0.94661 (4)	0.20807 (3)	0.0911 (2)	
O1	0.48628 (16)	0.2154 (2)	0.14770 (17)	0.0662 (7)	
O2	0.43563 (16)	0.6581 (2)	0.04692 (15)	0.0590 (6)	
O3	−0.01565 (17)	0.2636 (3)	0.02194 (17)	0.0791 (8)	
O4	0.02421 (14)	0.2088 (2)	0.16886 (14)	0.0513 (5)	
N1	0.40074 (18)	0.2682 (3)	0.1374 (2)	0.0543 (7)	
N2	0.12827 (16)	0.3153 (2)	0.13481 (16)	0.0389 (6)	
C1	0.2807 (2)	0.8543 (3)	0.1577 (2)	0.0550 (8)	
C2	0.2779 (2)	0.7113 (3)	0.1518 (2)	0.0476 (8)	
H2	0.2406	0.6620	0.1719	0.057*	
C3	0.3299 (2)	0.6404 (3)	0.1163 (2)	0.0424 (7)	
C4	0.3153 (2)	0.4858 (3)	0.0952 (2)	0.0429 (7)	
H4	0.2828	0.4793	0.0280	0.051*	
C5	0.4033 (2)	0.4004 (3)	0.1233 (2)	0.0425 (7)	
C6	0.5349 (2)	0.3220 (4)	0.1381 (2)	0.0584 (9)	
H6	0.5940	0.3139	0.1411	0.070*	
C7	0.4885 (2)	0.4401 (3)	0.1237 (2)	0.0462 (8)	
C8	0.5156 (2)	0.5831 (4)	0.1103 (3)	0.0598 (9)	
H8A	0.5432	0.6312	0.1689	0.072*	
H8B	0.5615	0.5784	0.0868	0.072*	
C9	0.3850 (2)	0.7190 (3)	0.0876 (2)	0.0468 (7)	
C10	0.3868 (2)	0.8620 (4)	0.0935 (2)	0.0605 (9)	
H10	0.4241	0.9122	0.0736	0.073*	
C11	0.3343 (3)	0.9314 (3)	0.1283 (3)	0.0635 (10)	
H11	0.3351	1.0276	0.1318	0.076*	
C14	0.1088 (2)	0.3832 (4)	−0.0252 (2)	0.0559 (9)	
H14A	0.0488	0.3422	−0.0430	0.084*	
H14B	0.1407	0.3343	−0.0543	0.084*	
H14C	0.1014	0.4786	−0.0439	0.084*	
C12	0.2510 (2)	0.4182 (3)	0.1294 (2)	0.0404 (7)	
C13	0.1637 (2)	0.3746 (3)	0.0763 (2)	0.0404 (7)	
C15	0.19718 (19)	0.3240 (3)	0.2260 (2)	0.0376 (7)	
C16	0.1986 (2)	0.2815 (3)	0.3088 (2)	0.0451 (7)	
H16	0.1481	0.2360	0.3103	0.054*	
C17	0.2775 (2)	0.3094 (4)	0.3884 (2)	0.0537 (8)	
H17	0.2801	0.2820	0.4444	0.064*	
C18	0.3536 (2)	0.3776 (4)	0.3872 (2)	0.0534 (8)	
H18	0.4057	0.3961	0.4422	0.064*	
C19	0.3518 (2)	0.4174 (3)	0.3050 (2)	0.0507 (8)	
H19	0.4027	0.4623	0.3042	0.061*	
C20	0.27401 (19)	0.3904 (3)	0.2236 (2)	0.0393 (7)	
C21	0.0393 (2)	0.2605 (3)	0.1012 (2)	0.0476 (8)	
C22	−0.0658 (2)	0.1391 (4)	0.1484 (2)	0.0542 (9)	
C23	−0.0521 (3)	0.0973 (5)	0.2429 (3)	0.0806 (13)	
H23A	−0.0023	0.0314	0.2682	0.121*	
H23B	−0.1082	0.0562	0.2389	0.121*	
H23C	−0.0371	0.1777	0.2817	0.121*	
C24	−0.0771 (3)	0.0154 (4)	0.0874 (3)	0.0829 (12)	
H24A	−0.0255	−0.0464	0.1173	0.124*	
H24B	−0.0793	0.0459	0.0302	0.124*	
H24C	−0.1338	−0.0320	0.0759	0.124*	
C25	−0.1444 (2)	0.2408 (5)	0.1069 (3)	0.0769 (12)	
H25A	−0.1527	0.2662	0.0466	0.115*	
H25B	−0.1304	0.3220	0.1449	0.115*	
H25C	−0.2005	0.1991	0.1028	0.115*	

### Atomic displacement parameters (Å^2^)

**Table:**

	*U*^11^	*U*^22^	*U*^33^	*U*^12^	*U*^13^	*U*^23^
Br1	0.1213 (4)	0.0499 (3)	0.1235 (4)	0.0187 (2)	0.0738 (3)	−0.0069 (2)
O1	0.0673 (15)	0.0465 (15)	0.0968 (19)	0.0139 (12)	0.0474 (14)	0.0101 (13)
O2	0.0687 (15)	0.0561 (15)	0.0683 (15)	−0.0004 (12)	0.0448 (13)	0.0146 (12)
O3	0.0632 (16)	0.114 (2)	0.0528 (16)	−0.0311 (15)	0.0189 (13)	0.0067 (15)
O4	0.0489 (12)	0.0553 (14)	0.0505 (12)	−0.0162 (11)	0.0227 (10)	0.0038 (10)
N1	0.0567 (16)	0.0364 (16)	0.082 (2)	0.0024 (13)	0.0422 (15)	0.0006 (14)
N2	0.0426 (13)	0.0335 (14)	0.0452 (14)	−0.0037 (11)	0.0237 (12)	0.0003 (11)
C1	0.062 (2)	0.0330 (19)	0.066 (2)	0.0045 (16)	0.0249 (18)	0.0017 (16)
C2	0.0547 (18)	0.0345 (18)	0.0562 (19)	−0.0033 (15)	0.0268 (16)	0.0012 (14)
C3	0.0484 (17)	0.0322 (17)	0.0484 (18)	−0.0027 (14)	0.0231 (15)	0.0042 (13)
C4	0.0460 (17)	0.0392 (18)	0.0475 (17)	−0.0070 (14)	0.0244 (15)	−0.0019 (14)
C5	0.0497 (18)	0.0348 (17)	0.0489 (18)	−0.0037 (14)	0.0272 (15)	−0.0040 (14)
C6	0.0510 (19)	0.060 (2)	0.071 (2)	0.0018 (18)	0.0331 (18)	0.0040 (18)
C7	0.0451 (17)	0.050 (2)	0.0492 (18)	−0.0044 (16)	0.0255 (15)	0.0002 (15)
C8	0.052 (2)	0.056 (2)	0.083 (2)	−0.0058 (17)	0.0392 (19)	0.0059 (19)
C9	0.0470 (17)	0.0425 (19)	0.0505 (18)	−0.0052 (15)	0.0212 (15)	0.0082 (15)
C10	0.062 (2)	0.043 (2)	0.077 (2)	−0.0082 (18)	0.0308 (19)	0.0171 (18)
C11	0.067 (2)	0.0293 (19)	0.084 (3)	−0.0055 (18)	0.024 (2)	0.0081 (17)
C14	0.063 (2)	0.059 (2)	0.051 (2)	−0.0106 (18)	0.0301 (17)	0.0022 (17)
C12	0.0488 (18)	0.0288 (16)	0.0482 (18)	0.0001 (13)	0.0257 (15)	0.0000 (13)
C13	0.0494 (17)	0.0288 (16)	0.0494 (18)	−0.0029 (14)	0.0279 (15)	0.0000 (13)
C15	0.0414 (16)	0.0247 (15)	0.0534 (19)	0.0016 (13)	0.0271 (15)	0.0002 (13)
C16	0.0476 (18)	0.0396 (18)	0.0522 (19)	0.0040 (14)	0.0259 (16)	0.0007 (15)
C17	0.061 (2)	0.053 (2)	0.052 (2)	0.0126 (18)	0.0289 (18)	0.0041 (16)
C18	0.0466 (18)	0.053 (2)	0.0486 (19)	0.0052 (16)	0.0106 (15)	−0.0094 (16)
C19	0.0513 (19)	0.048 (2)	0.052 (2)	0.0023 (16)	0.0223 (17)	−0.0009 (15)
C20	0.0404 (16)	0.0293 (15)	0.0478 (18)	0.0009 (13)	0.0191 (14)	−0.0047 (13)
C21	0.0484 (19)	0.045 (2)	0.049 (2)	−0.0041 (15)	0.0211 (17)	−0.0001 (15)
C22	0.0475 (19)	0.054 (2)	0.060 (2)	−0.0169 (17)	0.0229 (16)	0.0019 (17)
C23	0.072 (2)	0.095 (3)	0.072 (2)	−0.031 (2)	0.030 (2)	0.018 (2)
C24	0.090 (3)	0.061 (3)	0.105 (3)	−0.032 (2)	0.050 (3)	−0.020 (2)
C25	0.053 (2)	0.090 (3)	0.087 (3)	−0.004 (2)	0.031 (2)	0.005 (2)

### Geometric parameters (Å, °)

**Table:**

Br1—C1	1.887 (3)	C11—H11	0.93
O1—C6	1.342 (4)	C14—C13	1.487 (4)
O1—N1	1.403 (3)	C14—H14A	0.96
O2—C9	1.384 (4)	C14—H14B	0.96
O2—C8	1.436 (4)	C14—H14C	0.96
O3—C21	1.195 (4)	C12—C13	1.347 (4)
O4—C21	1.321 (4)	C12—C20	1.429 (4)
O4—C22	1.492 (3)	C15—C16	1.396 (4)
N1—C5	1.300 (4)	C15—C20	1.405 (4)
N2—C21	1.388 (4)	C16—C17	1.376 (4)
N2—C15	1.403 (4)	C16—H16	0.93
N2—C13	1.425 (3)	C17—C18	1.396 (5)
C1—C11	1.372 (5)	C17—H17	0.93
C1—C2	1.382 (4)	C18—C19	1.376 (4)
C2—C3	1.386 (4)	C18—H18	0.93
C2—H2	0.93	C19—C20	1.383 (4)
C3—C9	1.392 (4)	C19—H19	0.93
C3—C4	1.525 (4)	C22—C25	1.502 (5)
C4—C12	1.518 (4)	C22—C23	1.503 (5)
C4—C5	1.520 (4)	C22—C24	1.509 (5)
C4—H4	0.98	C23—H23A	0.96
C5—C7	1.417 (4)	C23—H23B	0.96
C6—C7	1.325 (5)	C23—H23C	0.96
C6—H6	0.93	C24—H24A	0.96
C7—C8	1.491 (4)	C24—H24B	0.96
C8—H8A	0.97	C24—H24C	0.96
C8—H8B	0.97	C25—H25A	0.96
C9—C10	1.383 (5)	C25—H25B	0.96
C10—C11	1.378 (5)	C25—H25C	0.96
C10—H10	0.93		
			
C6—O1—N1	107.3 (2)	C13—C12—C20	109.2 (2)
C9—O2—C8	113.6 (2)	C13—C12—C4	125.7 (3)
C21—O4—C22	120.0 (2)	C20—C12—C4	125.1 (3)
C5—N1—O1	105.8 (2)	C12—C13—N2	108.0 (2)
C21—N2—C15	129.1 (2)	C12—C13—C14	129.0 (3)
C21—N2—C13	122.4 (2)	N2—C13—C14	123.0 (3)
C15—N2—C13	108.5 (2)	C16—C15—N2	131.8 (3)
C11—C1—C2	121.5 (3)	C16—C15—C20	121.4 (3)
C11—C1—Br1	118.9 (3)	N2—C15—C20	106.9 (2)
C2—C1—Br1	119.6 (3)	C17—C16—C15	117.4 (3)
C1—C2—C3	121.0 (3)	C17—C16—H16	121.3
C1—C2—H2	119.5	C15—C16—H16	121.3
C3—C2—H2	119.5	C16—C17—C18	121.9 (3)
C2—C3—C9	117.2 (3)	C16—C17—H17	119.1
C2—C3—C4	121.0 (3)	C18—C17—H17	119.1
C9—C3—C4	121.0 (3)	C19—C18—C17	120.1 (3)
C12—C4—C5	110.4 (2)	C19—C18—H18	119.9
C12—C4—C3	113.6 (2)	C17—C18—H18	119.9
C5—C4—C3	115.6 (2)	C18—C19—C20	119.7 (3)
C12—C4—H4	105.4	C18—C19—H19	120.1
C5—C4—H4	105.4	C20—C19—H19	120.1
C3—C4—H4	105.4	C19—C20—C15	119.5 (3)
N1—C5—C7	111.7 (3)	C19—C20—C12	133.0 (3)
N1—C5—C4	119.2 (3)	C15—C20—C12	107.5 (2)
C7—C5—C4	128.4 (3)	O3—C21—O4	125.7 (3)
C7—C6—O1	111.7 (3)	O3—C21—N2	123.6 (3)
C7—C6—H6	124.2	O4—C21—N2	110.6 (3)
O1—C6—H6	124.2	O4—C22—C25	110.1 (3)
C6—C7—C5	103.6 (3)	O4—C22—C23	101.8 (3)
C6—C7—C8	130.1 (3)	C25—C22—C23	110.3 (3)
C5—C7—C8	126.3 (3)	O4—C22—C24	109.1 (3)
O2—C8—C7	110.2 (3)	C25—C22—C24	112.9 (3)
O2—C8—H8A	109.6	C23—C22—C24	112.1 (3)
C7—C8—H8A	109.6	C22—C23—H23A	109.5
O2—C8—H8B	109.6	C22—C23—H23B	109.5
C7—C8—H8B	109.6	H23A—C23—H23B	109.5
H8A—C8—H8B	108.1	C22—C23—H23C	109.5
C10—C9—O2	117.2 (3)	H23A—C23—H23C	109.5
C10—C9—C3	121.2 (3)	H23B—C23—H23C	109.5
O2—C9—C3	121.5 (3)	C22—C24—H24A	109.5
C11—C10—C9	121.0 (3)	C22—C24—H24B	109.5
C11—C10—H10	119.5	H24A—C24—H24B	109.5
C9—C10—H10	119.5	C22—C24—H24C	109.5
C1—C11—C10	118.1 (3)	H24A—C24—H24C	109.5
C1—C11—H11	121.0	H24B—C24—H24C	109.5
C10—C11—H11	121.0	C22—C25—H25A	109.5
C13—C14—H14A	109.5	C22—C25—H25B	109.5
C13—C14—H14B	109.5	H25A—C25—H25B	109.5
H14A—C14—H14B	109.5	C22—C25—H25C	109.5
C13—C14—H14C	109.5	H25A—C25—H25C	109.5
H14A—C14—H14C	109.5	H25B—C25—H25C	109.5
H14B—C14—H14C	109.5		
			
C6—O1—N1—C5	−0.5 (3)	C5—C4—C12—C20	59.5 (4)
C11—C1—C2—C3	0.4 (5)	C3—C4—C12—C20	−72.3 (4)
Br1—C1—C2—C3	−179.1 (2)	C20—C12—C13—N2	−0.9 (3)
C1—C2—C3—C9	0.4 (5)	C4—C12—C13—N2	179.0 (3)
C1—C2—C3—C4	−169.9 (3)	C20—C12—C13—C14	178.9 (3)
C2—C3—C4—C12	−8.4 (4)	C4—C12—C13—C14	−1.3 (5)
C9—C3—C4—C12	−178.3 (3)	C21—N2—C13—C12	179.9 (3)
C2—C3—C4—C5	−137.6 (3)	C15—N2—C13—C12	0.0 (3)
C9—C3—C4—C5	52.5 (4)	C21—N2—C13—C14	0.1 (4)
O1—N1—C5—C7	0.0 (3)	C15—N2—C13—C14	−179.8 (3)
O1—N1—C5—C4	171.0 (2)	C21—N2—C15—C16	0.4 (5)
C12—C4—C5—N1	26.0 (4)	C13—N2—C15—C16	−179.7 (3)
C3—C4—C5—N1	156.7 (3)	C21—N2—C15—C20	−179.0 (3)
C12—C4—C5—C7	−164.6 (3)	C13—N2—C15—C20	0.9 (3)
C3—C4—C5—C7	−33.9 (4)	N2—C15—C16—C17	−178.1 (3)
N1—O1—C6—C7	1.0 (4)	C20—C15—C16—C17	1.2 (4)
O1—C6—C7—C5	−0.9 (4)	C15—C16—C17—C18	0.0 (5)
O1—C6—C7—C8	179.3 (3)	C16—C17—C18—C19	−0.9 (5)
N1—C5—C7—C6	0.6 (4)	C17—C18—C19—C20	0.4 (5)
C4—C5—C7—C6	−169.4 (3)	C18—C19—C20—C15	0.8 (4)
N1—C5—C7—C8	−179.7 (3)	C18—C19—C20—C12	179.8 (3)
C4—C5—C7—C8	10.3 (5)	C16—C15—C20—C19	−1.7 (4)
C9—O2—C8—C7	84.3 (3)	N2—C15—C20—C19	177.8 (3)
C6—C7—C8—O2	145.6 (3)	C16—C15—C20—C12	179.1 (3)
C5—C7—C8—O2	−34.0 (4)	N2—C15—C20—C12	−1.4 (3)
C8—O2—C9—C10	110.5 (3)	C13—C12—C20—C19	−177.6 (3)
C8—O2—C9—C3	−73.3 (4)	C4—C12—C20—C19	2.6 (5)
C2—C3—C9—C10	−0.7 (5)	C13—C12—C20—C15	1.4 (3)
C4—C3—C9—C10	169.6 (3)	C4—C12—C20—C15	−178.4 (3)
C2—C3—C9—O2	−176.7 (3)	C22—O4—C21—O3	4.0 (5)
C4—C3—C9—O2	−6.3 (4)	C22—O4—C21—N2	−177.5 (2)
O2—C9—C10—C11	176.4 (3)	C15—N2—C21—O3	176.7 (3)
C3—C9—C10—C11	0.2 (5)	C13—N2—C21—O3	−3.2 (5)
C2—C1—C11—C10	−0.8 (5)	C15—N2—C21—O4	−1.8 (4)
Br1—C1—C11—C10	178.7 (3)	C13—N2—C21—O4	178.3 (2)
C9—C10—C11—C1	0.5 (5)	C21—O4—C22—C25	−63.4 (4)
C5—C4—C12—C13	−120.3 (3)	C21—O4—C22—C23	179.7 (3)
C3—C4—C12—C13	107.9 (3)	C21—O4—C22—C24	61.1 (4)

### Hydrogen-bond geometry (Å, °)

**Table:**

*D*—H···*A*	*D*—H	H···*A*	*D*···*A*	*D*—H···*A*
C14—H14A···O3	0.96	1.93	2.694 (4)	135
C24—H24B···O3	0.96	2.37	2.961 (5)	120
C11—H11···N1^i^	0.93	2.53	3.404 (4)	156

Symmetry codes:  (i) *x*, *y*+1, *z*.
